# Effects of oxidative and thermal stresses on stress granule formation in human induced pluripotent stem cells

**DOI:** 10.1371/journal.pone.0182059

**Published:** 2017-07-26

**Authors:** Freshteh Palangi, Samson M. Samuel, I. Richard Thompson, Chris R. Triggle, Mohamed M. Emara

**Affiliations:** 1 Neurological Disorders Research Center, Qatar Biomedical Research Institute, Hamad Bin Khalifa University, Qatar Foundation, Doha, Qatar; 2 Department of Pharmacology, Weill Cornell Medical College-Qatar, Qatar Foundation, Doha, Qatar; 3 Department of Virology, School of Veterinary Medicine, Cairo University, Cairo, Egypt; University of Texas at Austin Dell Medical School, UNITED STATES

## Abstract

Stress Granules (SGs) are dynamic ribonucleoprotein aggregates, which have been observed in cells subjected to environmental stresses, such as oxidative stress and heat shock (HS). Although pluripotent stem cells (PSCs) are highly sensitive to oxidative stress, the role of SGs in regulating PSC self-renewal and differentiation has not been fully elucidated. Here we found that sodium arsenite (SA) and HS, but not hydrogen peroxide (H_2_O_2_), induce SG formation in human induced (hi) PSCs. Particularly, we found that these granules contain the well-known SG proteins (G3BP, TIAR, eIF4E, eIF4A, eIF3B, eIF4G, and PABP), were found in juxtaposition to processing bodies (PBs), and were disassembled after the removal of the stress. Moreover, we showed that SA and HS, but not H_2_O_2_, promote eIF2α phosphorylation in hiPSCs forming SGs. Analysis of pluripotent protein expression showed that HS significantly reduced all tested markers (OCT4, SOX2, NANOG, KLF4, L1TD1, and LIN28A), while SA selectively reduced the expression levels of NANOG and L1TD1. Finally, in addition to LIN28A and L1TD1, we identified DPPA5 (pluripotent protein marker) as a novel component of SGs. Collectively, these results provide new insights into the molecular cues of hiPSCs responses to environmental insults.

## Introduction

Environmental stress induces swift response within the cell that leads to a timely adaptation of different regulatory processes such as chromatin remodeling, transcriptional regulation, and translational control that maximize the ability of cells to survive under these stressful conditions [1]. Cell translational arrest has been observed under different types of environmental stressors such as hypoxia [2], oxidative stress [3–5], heat shock (HS) [3, 6], and some viral infections [7–9]. Cessation of cell protein synthesis is caused by translation initiation inhibition that leads to rapid polysome disassembly and is associated with the activation of regulatory stress-response programs. These regulatory programs deal with stress conditions by reducing the expression of common housekeeping genes and increasing the expression of genes that repair stress-induced damage [10]. A consequence of such translation inhibition regulatory mechanism is the assembly of cytoplasmic nonmembranous structures known as stress granules (SGs).

SGs are sites of non-translating messenger ribounucleoproteins (mRNPs) aggregation under stress conditions. Those aggregates selectively store transcripts encoding housekeeping genes, but not those encoding stress-induced genes, as well as a wide variety of RNA binding proteins (RBPs) that are involved in many different metabolic and signaling pathways in the cell [11–13]. Indeed, SGs are involved in various stress-induced signaling cascades such as inflammatory signaling and stress-induced apoptotic signaling [14, 15]. Once the stress factor passes, SGs disassemble and the sorted mRNAs are released for active translation. It has been well established that SGs are present in a direct physical interaction with another type of RNA granules known as the processing (P) body (PBs) [16, 17], where both granules have been found to play a role in stress-induced translational arrest. SGs are formed as a consequence of eIF2α phosphorylation by one of the selective stress-activated kinases (HRI, PKR, PERK, or GCN2) [18–20], which in-turn inhibits translation initiation by reducing the availability of eIF2-GTP-tRNA_i_^Met^ ternary complex. However, this is not the only mechanism by which SGs are assembled. Other studies showed phospho-eIF2α independent mechanisms that induce translation inhibition and SG formation. For instance, an anti-inflammatory lipid mediator (15d-PGJ2) as well as xenobiotic agents pateamine A and hippuristanol have been identified as potent inhibitors of eukaryotic translation that powerfully induce SG formation via the interaction with eIF4A [21–23]. Moreover, other studies have reported that selective types of tiRNA, small fragments of tRNA, inhibit translation and induce SG formation by stress-induced disassembly of eIF4F [24–26]. The dynamic nature of these granules and their ability to selectively store and release specific mRNPs indicate their potential role in regulating gene expression, cell survival, and cell reprogramming.

Pluripotent stem cells (PSCs), Embryonic Stem Cells (ESCs) and induced pluripotent stem cells (iPSCs), have the unique capacity to self renew and differentiate into various types of cells [27–30]. The ability of PSCs to direct their fate to either self-renewal or differentiation is balanced by different extra- and intra-cellular factors. One of the factors that have been found to primarily control this phenomenon is the alteration in micro-environmental conditions [31]. For instance, at early stages of embryonic development, ESCs reside in a hypoxic environment, where the cells produce very low amounts of ATP, whereas during cell differentiation ATP production increases through mitochondrial oxidative phosphorylation, which in-turn generated Reactive Oxygen Species (ROS) such as O_2_ and H_2_O_2_ [32]. High levels of ROS subject the cells to oxidative stress, which forces the cell to posseses certain abilities to combat stress conditions. Furthermore, stress-signaling pathways were found to play a role in ESC differentiation process [33]. In addition, the forkhead box protein O1 (FoxO1) transcription factor, which plays an critical role in regulating stress response [34], was also found to be an important regulator of human (h)ESC pluripotency [35]. Indeed, FoxO1 and another member of the forkhead family, FoxD1, have been identified as mediators in reprogramming somatic cells to iPSCs [36]. Importantly the RBPs LIN28A [37–39] and LINE-1 type transposase domain containing 1 (L1TD1) [38], pluripotent protein markers for undifferentiated PSCs, were sequestered to SGs and PBs in hESCs. Moreover, other components of the ESC regulatory network, RELB [39] and Makarin1 (MKRN1) [40], are found to be component of SGs. Although, all the previously mentioned studies highlight the important role of stress in regulating pluripotency, there are still several questions that need to be addressed to elevate our knowledge of how PSCs regulate stress response programs to respond to different types of stressors.

In this study, we investigated the effect of two different types of stressors: oxidative stress (sodium arsenite (SA) and H_2_O_2_), and thermal stress (HS) on SG formation in human (h) iPSCs. Our results demonstrated that only SA and HS induce SG formation and selectively down-regulate pluripotent protein marker expression in hiPSCs. Screening for pluripotent proteins recruited to SGs confirms that LIN28A and L1TD1 are SG markers and identified DPPA5 as a new, but weakly recruited SG component. Altogether, our data demonstrate that not all stressors are capable of inducing SGs in hiPSCs, and the granules formed in stressed hiPSCs are typical SGs.

## Materials and methods

### Culturing and treatment of the cells

hiPSCs (IMR90-1) was purchased form WiCell. Cells were cultured on feeder free conditions using Matrigel (Corning) for cell attachment. Under these conditions hiPSC colonies were grown in mTeSR media (STEMCELL technologies) and maintained at 37^°^C in a CO_2_ incubator. For cell passage, cell colonies were dissociated to the appropriate size cell aggregate using non-enzymatic reagent (ReLeSR; STEMCELL technologies). Confluent cell colonies were treated with the indicated concentrations of SA, H_2_O_2_, and emetine (all purchased from Sigma-Aldrich) at 37^°^C in a CO_2_ incubator as previously described [5]. For HS treatment, plates were tightly sealed with Parafilm to ensure the maintenance of optimum CO_2_ levels within the plates and then incubated in the oven for the desired time and temperatures. Human Umbilical Vein Endothelial Cells (HUVEC) were purchased from ATCC and were grown and maintained in Dulbecco’s Modified Eagle’s Medium (DMEM; Invitrogen) supplemented with 10% FBS (Sigma-Aldrich), in a humidified atmosphere with 5% CO_2_ at 37°C.

### Antibodies

Mouse monoclonal antibodies to p70 S6 kinase (H-9) (SK1-hedles), PABP (10E10), eIF4A (N-19), rabbit polyclonal antibodies to eIF4G-(H-300), eIF4E (FL-217), and goat polyclonal antibodies to eIF3n and TIAR were all purchased from Santa Cruz; whereas the mouse monoclonal antibodies to G3BP were purchased from BD Biosciences. Mouse monoclonal antibodies to detect the pluripotent markers, SOX2, NANOG, and LIN28A were from Cell Signaling; whereas the KLF4 and DPPA5 were from Abcam and R&D, respectively. Anti-mouse and anti-rabbit secondary antibodies conjugated with horseradish peroxidase (HRP) were from Cell Signaling. Alexa Fluor 488, 555, and 647 conjugated secondary antibodies were purchased from Thermoscientific.

### Immuno-blotting

SDS-Polyacrylamide Gel Electrophoresis (SDS-PAGE) was performed and followed by immuno-blotting to detect the levels of peIF2α (S51), eIF2α, NANOG, OCT4, LIN28, KLF4, SOX2, L1TD1, DPPA5 and β-ACTIN as previously described [41]. Briefly, cell lysate protein (15–30μg) was separated on an SDS-PAGE gel and trans-blotted onto nitrocellulose membranes, blocked with 5% (w/v) non-fat dry milk or bovine serum albumin in Tris-buffered saline (TBS) containing 0.1% (v/v) Tween 20 and incubated in the relevant primary antibody (1:1000 dilution), at 4°C on a rocking platform, overnight. The next day, following incubation with HRP-linked secondary antibodies (1:2500) for 1h at room temperature on a rocking platform, the proteins were detected using enhanced chemiluminescence reagent (Sigma-Aldrich, Inc) and imaged on a Geliance P600 gel documentation system (PerkinElmer, Inc.). When probing for peIF2α (S51), the total eIF2α form was detected in the same blot after stripping. β-ACTIN was used as the loading control. The band densities of the western blot images obtained were then quantified using the basic Quantity One software (Biorad, Inc.).

### Immunofluorescence microscopy

In a 24 well plate, cover slips were pretreated with matrigel for 2h and pluripotent stem cells were cultured as mentioned above until they reach the appropriate size colonies (~60% colony confluency) after about three days from the passage. After treatment, cells were washed thrice with PBS, fixed in 4% para-formaldehyde for 15–30 minutes at room temp (RT) and then subsequently washed three times with TBST (TBS with 0.2% Tween). Fixed cells were then permeabilized using PBST (PBS with 0.2% Triton) for 10 minutes, washed three times with TBST, and incubated in blocking buffer (5% normal horse serum (Hyclone) in PBS) with continuous shaking for at least 1 hour at RT. After blocking, cells were incubated with continuous shaking overnight at 4^°^C with the desired primary antibodies, diluted in the blocking buffer; p70 S6 kinase, PABP, eIF4A, eIF4G-(H-300), eIF4E, eIF3n, TIAR, and G3BP were all diluted 1:200; DPPA5, LIN28A and L1TD1 were diluted 1:100, while SOX2, KLF4, and NANOG were diluted 1:1000. Afterwards, cells were washed 3 times with TBST, stained with appropriate secondary antibodies (1:2000 dilution) for 1h rocking at RT, and then washed three times with TBST. Finally, 0.5μg/ml Hoechst 33258 dye (Molecular probes) were added to the cells for nuclear staining. After washing with PBS, cover slips were mounted in polyvinyl mounting medium and the cells were viewed and photographed with Axio Ziess fluorescence microscope using 20X and 40X objectives. The images were merged and analyzed using Gimp (v.2.8) and Scribus (v.1.4).

### Stress granules quantification

In ~10 separate fields within each coverslip, cells containing stress granules were counted manually. Any cell more than 3 distinct and clear granules was considered as a stress granule forming cell. Dead cells or morphologically differentiated cells are excluded from the count. The percentage of cells with SGs was quantified by counting ~700 cells/experiment.

### RNA isolation and RT-PCR

IMR90-1 cells (80% confluence) were grown in 35 mm dish and treated with either 125 uM of SA, 250 uM of H_2_O_2_, or HS (42°C). Cells were washed three times with PBS, dissociated using ReLeSR, and then total cell RNA was extracted from treated or non-treated cells using RNeasy plus Mini RNA extraction Kit (QIAGEN) according to the manufacturer's protocol. cDNA was synthesized using the Superscript III first-strand cDNA synthesis kit (Invitrogen) according the manufacturer protocol and then used as a template for PCR reaction to qualitatively examine the gene expression of pluripotency genes. GAPDH was used as loading control. Sequences of the forward (F) and the reverse (R) primers to amplify each gene are listed in Table 1.

**Table 1:** Primers used in this study.

Gene name	Sequence
*hGAPDH*	F5’ACGACCACTTTGTCAAGCTCATTTC3’
	R5’GCAGTGAGGGTCTCTCTCTTCCTCT3’
*hSOX2*	F5’GGGAAATGGGAGGGGTGCAAAAGAGG3’
	R5’TTGCGTGAGTGTGGATGGGATTGGTG3’
*hOCT4*	F5’GACAGGGGGAGGGGAGGAGCTAGG3’
	R5’CTTCCCTCCAACCAGTTGCCCCAAAC3’
*hNANOG*	F5’CATGAGTGTGGATCCAGCTTG3’
	R5’CCTGAATAAGCAGATCCATGG3’
*hKLF4*	F5’CCCAATTACCCATCCTTCCT3’
	R5’ACGATCGTCTTCCCCTCTTT3’
*hL1TD1*	F5’TGATGTTTGAGGAGATGAGGGA3’
	R5’TGTTGCTGATGAAAGGTCGG3’
*hLIN28A*	F5’GTAAGCTGCACATGGAAGGG3’
	R5’CGCCTCTCACTCCCAATACA3’

## Results

### Sodium arsenite and heat shock, but not H_2_O_2_, induce SG assembly in human induced pluripotent stem cells

**Fig 1:**
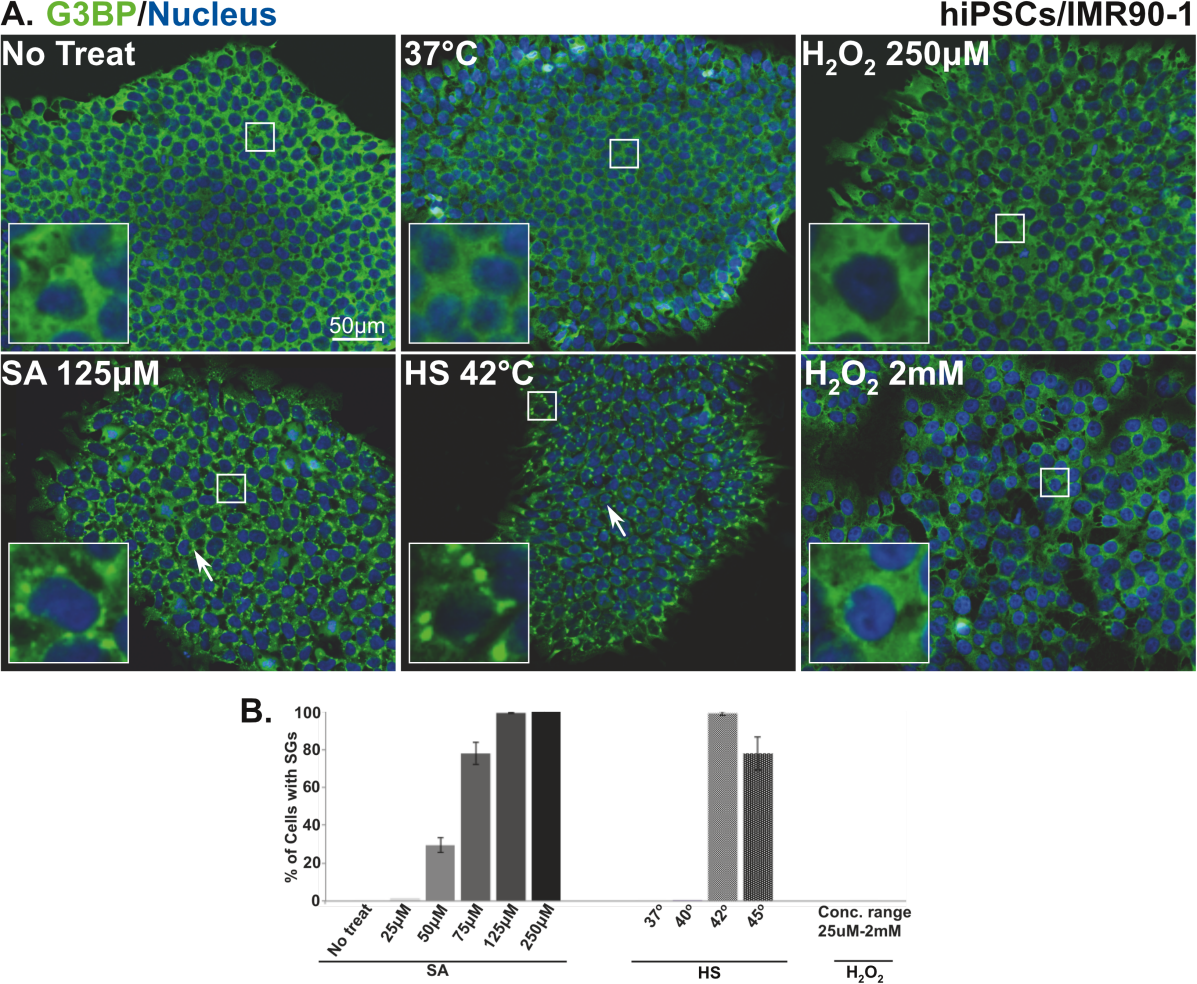
SG assembly in human induced pluripotent stem cells is stress specific. **(A)** Representative fluorescence microscopy images showing non-treated hiPSCs (No treat and 37^°^C), cells treated with 125μM sodium arsenite and H_2_O_2_ (250μM and 2mM) or subjected to heat shock (42^°^C) stained with the robust SG marker (G3BP (green)). Nucleus is stained in blue (Hoechst). Insets show magnified views of SGs. White arrows indicate SGs. Scale bar indicates 50μm. **(B)** Percentage of hiPSCs with G3BP positive SGs after 1h treatment with the indicated concentrations of sodium arsenite, or concentration range of H_2_O_2_ (25, 50, 100, 250, 500μM, 1mM, and 2 mM) or subjected to heat shock at the indicated temperatures. The average percentage of cells with SGs is shown. Error bars indicate the ± standard deviation from 3 independent experiments.

To determine whether different types of stressors induce SG formation in pluripotent stem cells, hiPSCs (IMR90-1) were treated with different concentrations of SA or H_2_O_2_, or subjected to HS at different temperatures. Each treatment was incubated for 1h, then cells were fixed, stained with the robust SG marker (G3BP), and quantified for SG formation using fluorescence microscopy. Out of the three stressors, only SA and HS only induce the formation of SGs (Fig 1A; white arrows), as compared to non-treated cells that showed no granules (Fig 1A and 1B). SA treatment induced SG formation in a concentration dependent manner (Fig 1B). At 50μM, 25% of cells were positive for SGs and as the concentrations of SA increase, the number of cells showing SGs also progressively increases to reach 80% (75μM concentration) and then 100%, a stationary phase, with higher concentrations of 125 and 250μM (Fig 1B). Conversely, a different pattern of SG formation was observed in HS treated cells. Although, no granules were detected in cells incubated at near physiological incubation temperature (37^°^C) or subjected to mild HS (40^°^C) treatment, 100% of the cells formed SGs after exposure to 42^°^C (Fig 1A and 1B). Interestingly, elevating the temperature to 45^°^C had an adverse effect on SG formation as the number of cells bearing SGs decreases to 80% (Fig 1B). Surprisingly, hiPSCs treated with H_2_O_2_ exhibited no SG formation even at concentrations up to 2mM (Fig 1A and 1B). This is in contrast to U2OS [5] or HUVEC (S1 Fig) cells that formed SGs after H_2_O_2_ treatment. These data indicate that not all stressors are capable of inducing SGs in hiPSCs.

A SA concentration of 125μM proved to be the lowest to induce SG formation in all treated cells after 1h of treatment. Similarly, shocking the cells with a temperature of 42^°^C for 1h was the optimum for SG induction. To ensure that these conditions did not affect the stem cell colony morphology, which is the visual characteristic of stem cell pluripotency, we tested the shape and size of non-treated and treated hiPSCs colonies using phase contrast microscopy. Typical pluripotent stem cell colony morphology was observed in non-treated as well as treated cells (S2 Fig). The colonies appear with distinct, circular, and homogenous borders and are composed of small, compact, uniform, and healthy cells that are densely packed together (S2 Fig; inset). These results indicate that these conditions did not alter pluripotent cell morphology and therefore were used for all subsequent experiments.

### Granules formed in human induced pluripotent stem cells are canonical stress granules

**Fig 2:**
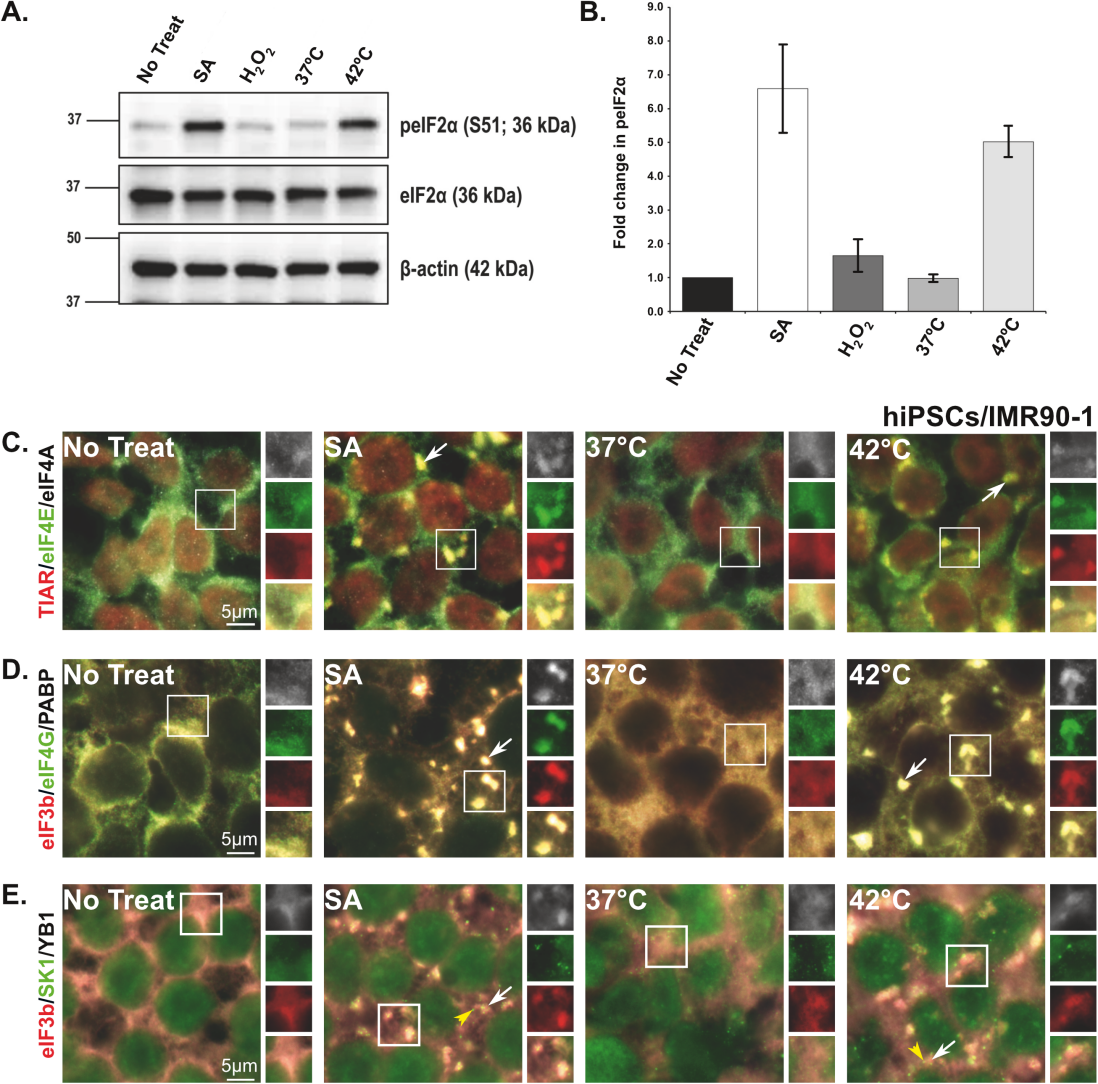
Sodium arsenite and heat shock treatments induce eIF2α phosphorylation and localize stress granule proteins in human induced pluripotent stem cells. **(A and B)** SA and HS treatments induce eIF2α phosphorylation and SG formation in hiPSCs. **(A)** Representative Immuno-blots that show the levels of peIF2α (Ser51) and eIF2α in non-treated cells (No treat and 37^°^C) or cells treated with 125μM sodium arsenite and 250μM H_2_O_2_, or subjected to heat shock **(**42^°^C). **(B)** Bar graph representing the ratio of (normalized against β-actin loading controls) peIF2α (Ser51)/eIF2α for each treatment. Values are expressed as mean ± SEM from 4–5 experiments. Asterisks indicate a statistically significant change, *p<0.05, **p<0.001, and ***p<0.0001. **(C-E)** Representative fluorescence microscopy images showing hiPSCs treated with sodium arsenite (125μM), subjected to heat shock (42^°^C), or left untreated (No treat; 37^°^C), and stained with SG markers **(C)** TIAR (red)/eIF4E (green)/ eIF4A (white), **(D)** eIF3b (red)/eIF4G (green)/PABP (white), and **(E)** eIF3b (red)/YB1 (white) or PB marker (SK1, green). Insets are reproduced at the right as replicate views of SGs showing each marker separately and the merged view (yellow). Scale bar indicates 5μm.

One of the well-known upstream events that is associated with SG formation induced by SA, HS [42], and H_2_O_2_ [5] is the phosphorylation of eIF2α (peIF2α). In the case of SA and HS treatments, the assembly of SGs is dependent on eIF2α phosphorylation, whereas the induction of these granules is peIF2α-independent in cells treated with H_2_O_2_. Therefore, it was of interest to test if this phenomenon also occurs in stressed hiPSCs. Using immuno-blot analysis, we analyzed the phosphorylation of eIF2α in cells treated with SA (125μM), HS (42^°^C), or H_2_O_2_ (250μM). No eIF2α phosphorylation was detected in non-treated cells or cells incubated at 37 ^o^C (Fig 2A and 2B). Notably, in cells treated with H_2_O_2_, which did not induce SG formation (Fig 1A), the levels of peIF2α were comparable to those of non-treated cells (Fig 2B), demonstrating that H_2_O_2_ is incapable of inducing either phosphorylation of eIF2α or SGs in hiPSCs. In contrast, and consistent with our SG data (Fig 1), both SA and HS that induce SG formation in hiPSCs, significantly induce eIF2α phosphorylation (Fig 2A and 2B). These data indicate that stressors such as SA and HS that induce eIF2α phosphorylation result in the formation of SGs in hiPSCs.

It has been previously reported that SG formation differs depending on the cell and nature of stress [43]. To determine whether SGs formed in hiPSCs have the same molecular signature as canonical SGs, the components of SGs were analyzed using a subset of known SG markers, TIAR, eIF4E, eIF4A (Fig 2C); eIF3B, eIF4G, and PABP (Fig 2D). As expected in non-treated cells or cells incubated at 37^°^C, all SG protein markers were normally distributed within the cytoplasm or the nucleus (Fig 2C and 2D). In contrast, in hiPSCs treated with SA or subjected to HS, these markers were recruited to SGs (Fig 2C and 2D; white arrows). All these proteins showed similar levels of recruitment with both stressors as indicated by fluorescence signal intensity (Fig 2, SA and 42^°^C; enlarged boxes). These results indicate that the formation of SGs in hiPSCs subjected to SA and HS exhibit similar characteristics to those of canonical SGs.

It has been established that the existence of SGs is associated with a direct physical interaction with another type of RNA granule known as P-bodies (PBs) [44]. In another approach to confirm SG formation in hiPSCs, the physical interaction between PBs and SGs were tested using the PB marker (SK1-hedles). As shown in Fig 2E, in hiPSCs subjected to SA or HS, PBs (yellow arrowhead) were observed in close association with SGs (Fig 2E; white arrows). Interestingly, the presence of PBs in juxtaposition to SGs in HS hiPSCs has not been documented in immortalized human cell lines (U2OS and Hela cells) [44] indicating that this is may be a unique property of hiPSCs.

**Fig 3:**
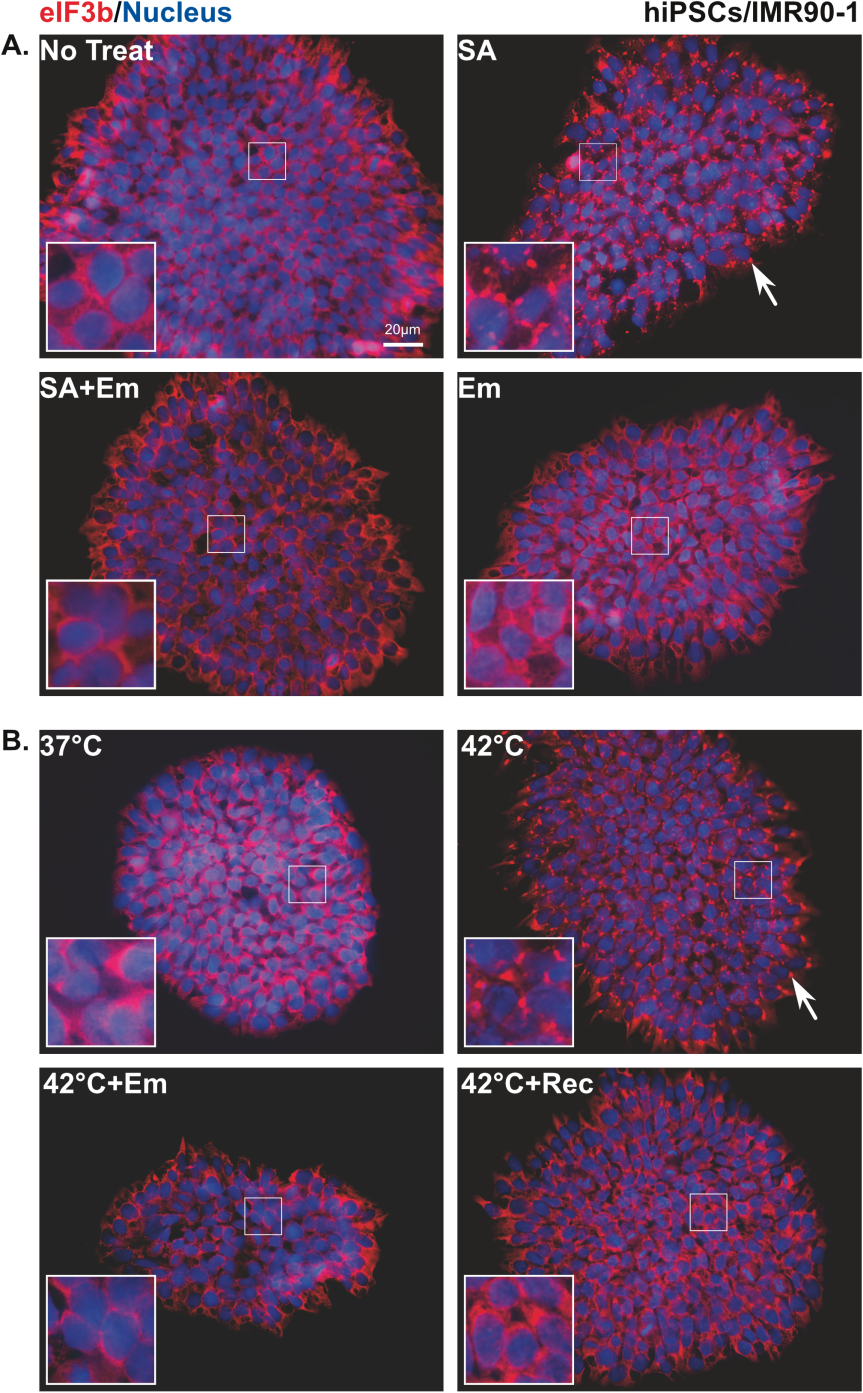
Emetine inhibits stress granule formation in stressed human induced pluripotent stem cells. **(A**) Cells treated with sodium arsenite or **(B)** heat shock (42^°^C) followed by emetine treatment (SA+Em; 42 ^o^C+Em) or recovered from HS (42 ^o^C+Rec) and stained with SG marker eIF3b (red). Nucleus is stained in blue (Hoechst). Insets show magnified views of SGs. White arrows indicate SGs and yellow arrowhead indicate PBs. Scale bar indicates 20μm.

To further confirm that the observed granules are *bona fide* SGs, we exposed stressed or non-stressed cells to the metabolic inhibitor emetine (Em) that is known to inhibit SG formation by interfering with polysome disassembly [45]. SGs (Fig 3A and 3B; white arrows) were detected in stressed cells, whereas after treating these cells with Em for 90 min (Fig 3A; SA+Em and Fig 3B; 42^°^C+Em), the granules were completely abolished and cells were comparable to non-treated ones (Fig 3A; No treat and Fig 3B; 37^°^C) or those treated with Em alone (Fig 3A; Em). To ensure that the SG formation in cells subjected to HS is solely due to a temperature effect, we returned the thermally shocked cells to normal environmental conditions at 37^°^C for 60 min. As shown in Fig 3B (42^°^C+Rec), reverting hiPSCs back to 37^°^C disassembled SGs. Altogether, these data demonstrate that the granules formed in stressed hiPSCs are typical SGs in terms of size, number, and types of protein recruited, despite differences in the characteristics of the granules induced by HS treatment.

### Pluripotent proteins are selectively down-regulated after stress conditions

**Fig 4:**
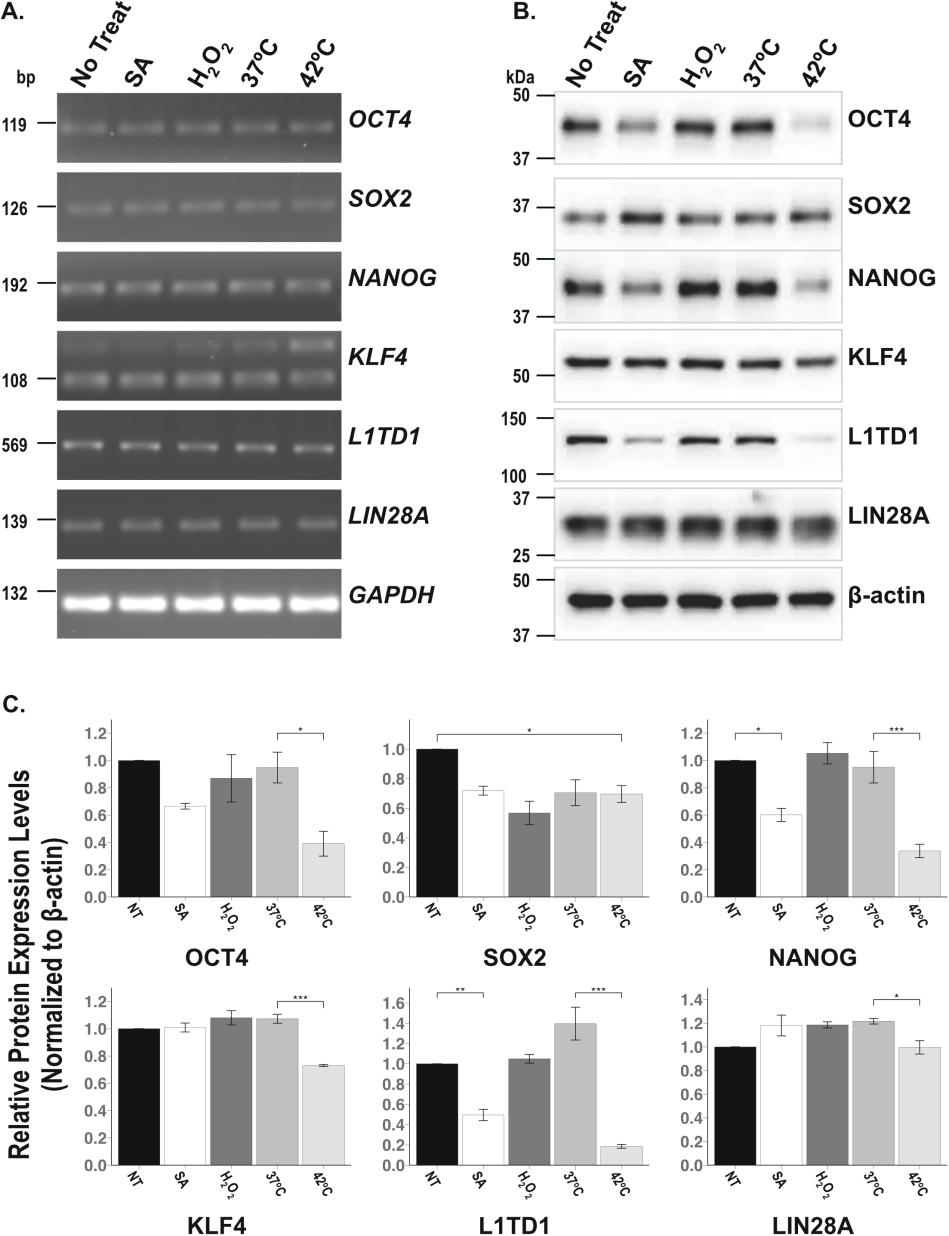
Down-regulation of some pluripotent protein expression is stress specific. (**A**) RT-PCR on mRNA extracted from non-treated hiPSCs (No treat and 37^°^C) or cells subjected to sodium arsenite, H_2_O_2_, and heat shock, to detect pluripotent genes, *OCT4*, *SOX2*, *NANOG*, *KLF4*, *L1TD1*, and *LIN28A*. GAPDH is used as a loading control. (**B**) Representative Immuno-blots illustrating the protein levels of OCT4, SOX2, NANOG, KLF4, L1TD1, and LIN28A. (**C**) Bar graphs represent the levels (normalized with β-actin loading controls) of the tested proteins. Asterisks indicate statistical significance p values; *p<0.05, **p<0.001 and ***p<0.0001. The values are expressed in mean ± SEM from 3 experiments.

The exposure of mouse ESCs to low concentration of SA (6μM) for 16h down regulates the expression of pluripotent proteins, SOX2, OCT4, and NANOG [46]. To determine whether different stresses regulate pluripotent proteins expression in hiPSCs, total RNA was extracted from non-treated cells or cells subjected to SA, H_2_O_2_ or HS, and a qualitative RNA analysis of six endogenous pluripotent protein markers, *OCT4*, *SOX2*, *NANOG*, *KLF4*, *L1TD1*, *and LIN28A*, was performed using RT-PCR. As shown in Fig 4A, cells subjected to any of the three stressors expressed pluripotent genes in levels comparable to non-treated cells. To confirm these results the pluripotent proteins extracted from treated or non-treated cell lysates were assessed by immuno-blot analyses. The expression levels of each protein were quantified using a densitometer and normalized to the levels of non-treated cells. Interestingly, in contrast to pluripotent protein gene expression levels, SA and HS selectively down-regulate the expression of pluripotent protein markers (Fig 4B and 4C). In SA treated cells, NANOG and L1TD1 are the only pluripotent protein markers that were significantly decreased compared to non-treated cells, whereas in cells subjected to 42^°^C, the expression of all other pluripotent protein markers was significantly decreased (Fig 4B and 4C). Conversely, H_2_O_2_, which did not induce SGs and has no effect on pluripotent gene expression, also failed to alter the expression levels of the pluripotent protein markers tested except for SOX2, which was found to be down-regulated (Fig 4B and 4C). These data classify SA and HS as SG inducers that selectively down-regulate pluripotent protein expression under stress conditions.

### Pluripotent protein markers are selectively recruited to SGs under stress conditions

Since only SA and HS, were able to induce SGs and selectively affect the protein expression of some pluripotent protein markers under stress conditions (Fig 4), it was of interest to perform a pilot screen to test the ability of pluripotent protein markers to aggregate into SGs. It is known that RBPs are the main proteins that are recruited to SGs [47], therefore the first step was to analyze pluripotent protein markers for the presence of RNA binding domains (RBD). Based on published data, we selected 36 proteins that are known to be the most common and well known human pluripotent markers [48] and screened them to identify possible RBD (Table 2) using the RNA-Binding Protein DataBase (RBPD) http://rbpdb.ccbr.utoronto.ca/ [49]. Of these, we identified only three proteins, LIN28A, L1TD1, and DPPA5, which harbor RBD. LIN28A and L1TD1, which have previously been shown to co-localize in SGs [38], contain the CCCH zinc finger (ZF) RBD and RNA Recognition motif (RRM) domain, respectively, whereas the K homology (KH) domain is recognized as the RBD of DPPA5 (Table 2).

**Table 2:** Analysis of pluripotent stem cell proteins for the presence of RNA binding domains.

Human Stem Cell marker	RNA binding domain			Human Stem Cell marker	RNA binding domain			Human Stem Cell marker	RNA binding domain		
	RRM	KH	ZF		RRM	KH	ZF		RRM	KH	ZF
CD324				FoxD3				Sall4			
CD90				GCNF				SCF			
CD117				HMGA2				Sox2			
CD324				KLF4				SSEA-1			
CD90				KLF4				SSEA-3			
CD117				LEF1				SSEA-4			
Cripto				**L1TD1**				Stat3			
Dppa2				**LIN28**				TBN			
Dppa3				NAC1				TRA-1-60			
Dppa4				Nanog				TRA-1-81			
**Dppa5**				Oct-3/4				UTF1			
ECAT9				Rex1				ZFX			

**Fig 5:**
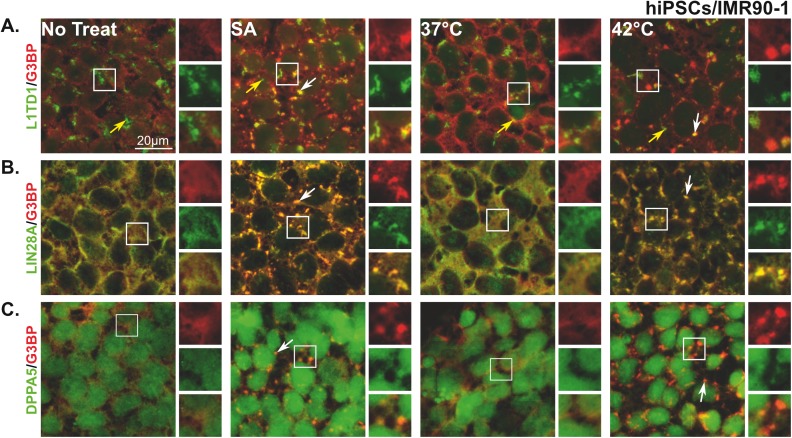
Pluripotent RNA binding proteins, L1TD1, LIN28A, and DPPA5 are components of SGs. Representative fluorescence microscopy images showing non-treated hiPSCs (No treat and 37^°^C) and cells treated with sodium arsenite (125μM) or subjected to heat shock (42^°^C) stained with the SG marker (G3BP (red)) and different pluripotent markers **(A)** L1TD1, **(B)** LIN28A, or **(C)** DPPA5 (green). At the right of each panel, insets show magnified views of SGs in individual and merged channels (yellow). White arrows indicate SGs and yellow arrows indicate L1TD1 aggregates. Scale bar indicates 20μm.

We next investigated whether the identified proteins in our screen are components of the SA and/or HS hiPSC SGs. Cells were left untreated or treated with either SA or HS (42^°^C) and then stained with antibodies directed against LIN28A, L1TD1, and DPPA5 as well as the SG marker, G3BP. Since we did not identify SOX2, NANOG, or KLF4 as RBPs, we stained them as negative RBP controls. As expected pluripotent protein markers that have no RBD were not recruited to SGs (S3 Fig). However, the three pluripotent RBPs (LIN28A, L1TD1, and DPPA5) co-localized with the SG marker, G3BP, in hiPSCs treated with SA or subjected to HS (Fig 5A–5C). Interestingly, in non-treated cells, L1TD1 appears as aggregates, which differ in shape and size to “standard” SGs (Fig 5A; No treat and 37^°^C; yellow arrows). Some of these aggregates, co-localize with G3BP SGs (white arrows), whereas others do not (Fig 5A; SA and 42^°^C). In contrast, LIN28A is highly recruited to SGs (Fig 5B, white arrows), as shown by its strong co-localization with G3BP (Fig 5B, yellow color). Staining stressed hiPSCs for DPPA5 demonstrates that the protein is weakly co-localized with G3BP in SGs (Fig 5C). These data confirm that pluripotent protein markers LIN28A and L1TD1 are part of hiPSC SGs formed as a result of SA or HS treatment and introduce DPPA5 as a novel component of SGs.

## Discussion

In the present study, we found that hiPSCs are able to induce SG formation depending on the insult used. Our major findings are (i) SA and HS treatments, but not H_2_O_2_, were able to aggregate core SG components and promote eIF2α phosphorylation in stressed hiPSCs, (ii) the assembled granules exhibit typical SGs features, including shape, size, number, composition, and disassembly process, (iii) RNA binding pluripotent markers LIN28A, L1TD1, and DPPA5, but not non-RNA binding pluripotent proteins (SOX2, KLF4, and NANOG), are selectively recruited to SGs in stressed hiPSCs, and (iv) DPPA5 is a novel component of SGs. To our knowledge this is the first attempt to compare the effect of different stresses on hiPSCs and investigate the characteristics of SGs on those cells.

SG formation is a very well recognized stress response phenomenon that is observed in cells subjected to different forms of environmental insult [12, 43, 47]. This phenomenon has been observed in a wide range of mammalian cells [12, 43], as well as yeast [50–52] and *Drosphila* cells [53–55] indicating that the assembly of these granules is evolutionary conserved amongst different species and hence plays a significant role in cell stress response. Despite being a common cell response, the formation and the composition of these granules differs among species, between cell types, and according to the stress types and impact. In *Drosophila*, granules formed in response to both HS and SA treatments resemble those formed in mammalian cells [53]. However, the composition of *Saccharomyces cerevisiae* granules formed after glucose deprivation lacks two major SGs components, eIF3 and 40S ribosomal subunits, but contain eukaryotic translation initiation factors eIF4E, eIF4G and Pab1p and thus called EGP bodies [56]. Interestingly, this was not the case when the same cells were treated with 1,6-hexanediol, [52] or subjected to HS [57], which remarkably induce canonical SGs.

It has been postulated that ESCs have higher tolerance to stress than differentiated cells, indicating that ESCs are equipped with various mechanisms to protect them from adverse environmental conditions [58]. The formation of SGs in stressed PSCs may be one of these protective mechanisms during development. This assumption is supported by the fact that SGs are able to selectively sequester and protect mRNA from degradation, which will eventually be required for ESC development [59]. The availability of maternally contributed mRNAs is a crucial step during embryonic development, because it initiates embryogenesis [60]. Moreover, the SG forming protein, TIAR, has been found to be essential in protecting *C*. *elegans* germ cells and embryos from HS insult [61]. Our data supported the proposition that SGs are a protection center for PSCs, where the pluripotent protein markers, LIN28A, L1TD1, and DPPA5 are sequestered to those granules during stress conditions, however, after recovery, SGs were completely disassembled. This data is consistent with others that show the recruitment of LIN28A and L1TD1 to SGs in stressed ESCs [38] and iPSCs [39]. The absence of other PPMs from SGs does not exclude the possibility that the RNA encoding those proteins may be the ones recruited to SGs. Our data illustrates that pluripotent gene expression is not altered after stress treatment, whereas the expression of some PPMs, such as NANOG, is significantly down-regulated, indicating that the mRNA encoding these proteins is either degraded or sequestered to SGs. In support of this hypothesis, Tan *et al*. [62] reported that *NANOG* mRNA harbors a full consensus AU-rich (ARE) element (AUUUAAUUUA) in its 3’ UTR and binds to Poly-(rC) Binding Protein 1, which was documented to be recruited to SGs and PBs [63]. Interestingly, ARE elements containing mRNAs were found to be highly recruited to SGs [47]. Importantly, TIAR, one of the main limiting factors of SG formation, specifically binds to this canonical AUUUA motif located at 3’UTR ARE elements of mRNAs [64]. Therefore, it is logical to assume that TIAR binds to NANOG mRNA and/or other ARE PPMs and sequesters it to SGs. Further experiments are needed to confirm this hypothesis.

To our surprise, hiPSCs did not assemble SGs in response to H_2_O_2_ treatment. This could be due to the high expression of glutathione peroxidases (Gpxs), antioxidant enzymes that detoxify H_2_O_2_ and protect PSCs from oxidative damage [65]. Indeed, undifferentiated iPSCs showed elevated levels of glutathione and significant up-regulation of several H_2_O_2_ scavengers including Gpx2 and GSTA2 as compared to differentiated cells [66]. In support to this notion, during the differentiation of mouse and hESCs, Gpx2 expression was down-regulated, indicating the important role of Gpx2 in maintaining the pluripotent status of stem cells [58]. Moreover, several studies demonstrated that undifferentiated hESCs show higher resistance to H_2_O_2_ as compared to differentiated cells [67–69]. Consistently, our results showed that H_2_O_2_ induces SGs in HUVEC (S1 Fig) and U2OS cells [5] but not hiPSCs. Therefore, it is conceivable that PSCs possess sufficient resistance against H_2_O_2_ insult to maintain the cells in its pluripotent status for a certain period of time without the need to induce SG formation.

In summary, our data suggest that hiPSCs are capable of inducing SG formation when subjected to adverse environmental conditions. These granules are stress-specific and stimulate the recruitment of selective PPMs. More studies are warranted in order to address why SGs are assembled in stressed PSCs. It is possible that SGs selectively sequester different classes of mRNAs and proteins that regulate pluripotency, which in turn lead to the regulation of signaling molecules that control stem cell fate. Thus SGs may be a switch between self-renewal and differentiation.

## Supporting information

S1 Fig H_2_O_2_ induce SG assembly in HUVEC. Fluorescence microscopy images showing **(A)** NT HUVEC (No treat) or **(B)** HUVEC treated with 1mM H_2_O_2_ and stained with the robust SG markers (G3BP (red)). Nucleus is stained in blue (Hoechst). White arrows indicate SGs.
(TIFF)

S2 Fig Analysis of hiPSC morphology after SA, HS, and H_2_O_2_ treatments. Phase contrast microscopy showing the morphology of **(A and C)** non-treated hiPSC colonies (No treat and 37^°^C) or colonies treated with **(B)** 125μM SA **(D)** HS **(**42^°^C) **(E)** 250μM H_2_O_2_, and **(F)** 2mM H_2_O_2_. Large insets show magnified views of cells.
(TIFF)

S3 Fig Non RNA binding pluripotent protein markers are not recruited to SGs under stress conditions. Representative fluorescence microscopy images showing hiPSCs treated with 125μM SA, subjected to HS (42^°^C), or left untreated (No treat; 37^°^C), and stained with SG markers TIAR (red) and different pluripotent markers **(A)** SOX2, **(B)** KLF4, or **(C)** NANOG (green). At the right of each panel, insets show magnified views of SGs in individual and merged channels (yellow).
(TIFF)
