# Supplementary figures and images for: Effects of oxidative and thermal stresses on stress granule formation in human induced pluripotent stem cells
Source: PLoS One. 2017 Jul 26;12(7):e0182059. doi: 10.1371/journal.pone.0182059 (PMC5528897; doi:10.1371/journal.pone.0182059)

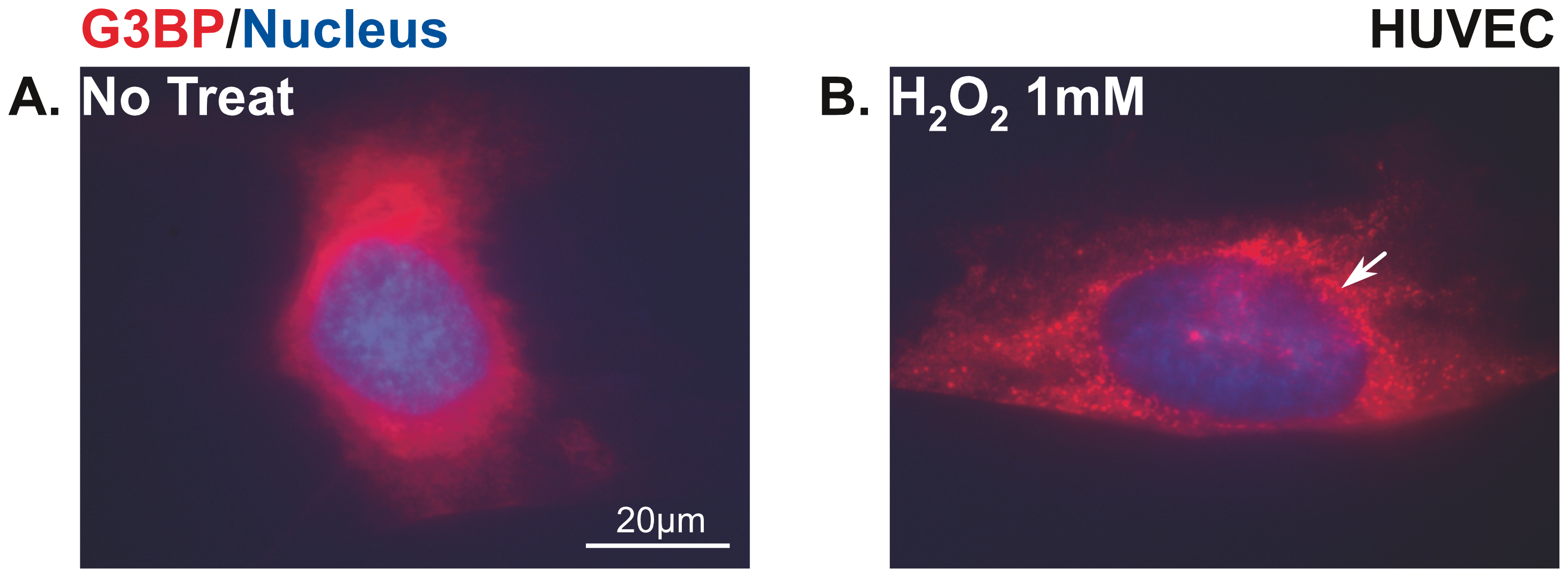

Supplement: S1 Fig — Fluorescence microscopy images showing (A) NT HUVEC (No treat) or (B) HUVEC treated with 1mM H2O2 and stained with the robust SG markers (G3BP (red)). Nucleus is stained in blue (Hoechst). White arrows indicate SGs. (TIFF) [file pone.0182059.s001.tiff]

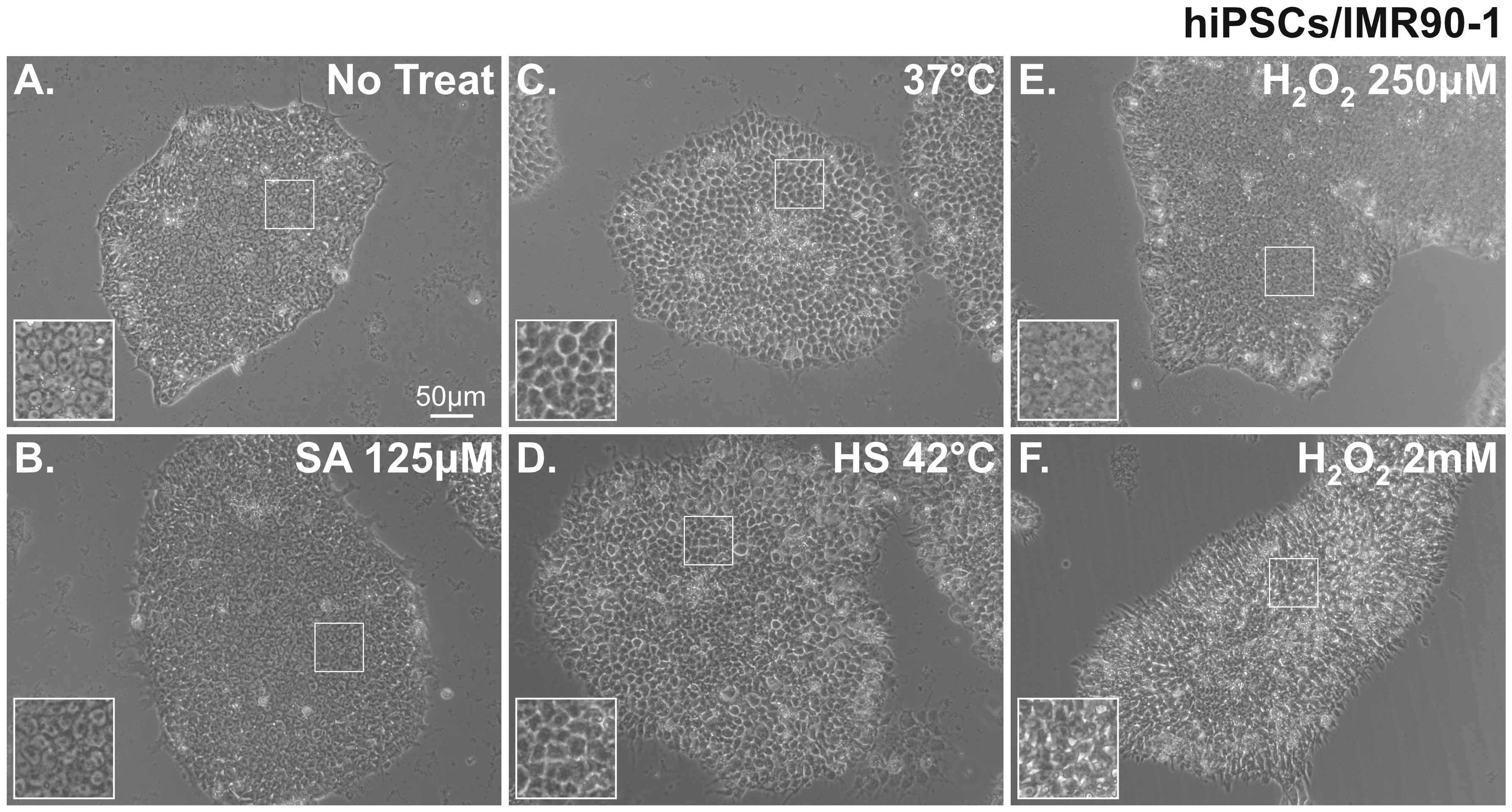

Supplement: S2 Fig — Phase contrast microscopy showing the morphology of (A and C) non-treated hiPSC colonies (No treat and 37°C) or colonies treated with (B) 125μM SA (D) HS (42°C) (E) 250μM H2O2, and (F) 2mM H2O2. Large insets show magnified views of cells. (TIFF) [file pone.0182059.s002.tiff]

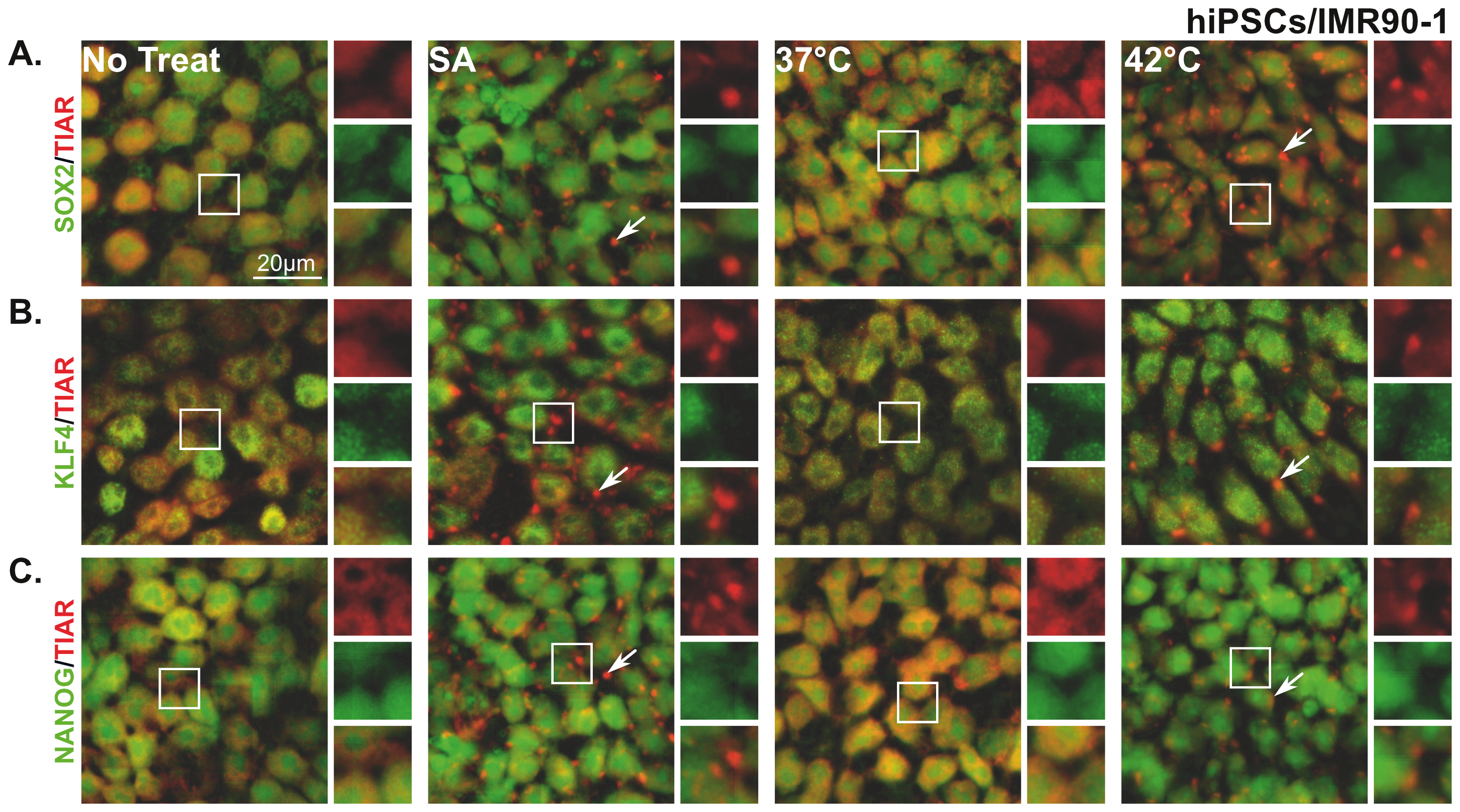

Supplement: S3 Fig — Representative fluorescence microscopy images showing hiPSCs treated with 125μM SA, subjected to HS (42°C), or left untreated (No treat; 37°C), and stained with SG markers TIAR (red) and different pluripotent markers (A) SOX2, (B) KLF4, or (C) NANOG (green). At the right of each panel, insets show magnified views of SGs in individual and merged channels (yellow). (TIFF) [file pone.0182059.s003.tiff]
